# The Effect of UVB Irradiation and Oxidative Stress on the Skin Barrier—A New Method to Evaluate Sun Protection Factor Based on Electrical Impedance Spectroscopy

**DOI:** 10.3390/s19102376

**Published:** 2019-05-23

**Authors:** Aura Rocio Hernández, Bibiana Vallejo, Tautgirdas Ruzgas, Sebastian Björklund

**Affiliations:** 1 Department of Pharmacy, Universidad Nacional de Colombia, Bogota 1101, Colombia; arhernandezc@unal.edu.co (A.R.H.); bmvallejod@unal.edu.co (B.V.); 2 Department of Biomedical Science, Malmö University, SE-205 06 Malmö, Sweden; tautgirdas.ruzgas@mau.se; 3 Biofilms—Research Center for Biointerfaces, Malmö University, SE-205 06 Malmö, Sweden

**Keywords:** oxidative stress, UVB irradiation, sun protection factor, cosmetic sunscreen, stratum corneum, epidermis, catalase, hydrogen peroxide, azide

## Abstract

Sunlight is vital for several biochemical processes of the skin organ. However, acute or chronic exposure to ultraviolet radiation (UVR) has several harmful effects on the skin structure and function, especially in the case of the failing function of antioxidative enzymes, which may lead to substantial tissue damage due to the increased presence of reactive oxygen species (ROS). The aim of this work was to investigate the combined effect of ultraviolet B (UVB) irradiation and oxidative stress on the skin barrier integrity. For this, we employed electrical impedance spectroscopy (EIS) to characterize changes of the electrical properties of excised pig skin membranes after various exposure conditions of UVB irradiation, oxidative stress, and the inhibition of antioxidative enzymatic processes. The oxidative stress was regulated by adding hydrogen peroxide (H_2_O_2_) as a source of ROS, while sodium azide (NaN_3_) was used as an inhibitor of the antioxidative enzyme catalase, which is naturally present throughout the epidermis. By screening for the combined effect of UVB and oxidative stress on the skin membrane electrical properties, we developed a new protocol for evaluating these parameters in a simple in vitro setup. Strikingly, the results show that exposure to extreme UVB irradiation does not affect the skin membrane resistance, implying that the skin barrier remains macroscopically intact. Likewise, exposure to only oxidative stress conditions, without UVB irradiation, does not affect the skin membrane resistance. In contrast to these observations, the combination of UVB irradiation and oxidative stress conditions results in a drastic decrease of the skin membrane resistance, indicating that the integrity of the skin barrier is compromised. Further, the skin membrane effective capacitance remained more or less unaffected by UVB exposure, irrespective of simultaneous exposure of oxidative stress. The EIS results were concluded to be associated with clear signs of macroscopic tissue damage of the epidermis as visualized with microscopy after exposure to UVB irradiation under oxidative stress conditions. Finally, the novel methodology was tested by performing an assessment of cosmetic sunscreen formulations with varying sun protection factor (SPF), with an overall successful outcome, showing good correlation between SPF value and protection capacity in terms of skin resistance change. The results from this study allow for the development of new skin sensors based on EIS for the detection of skin tissue damage from exposure to UVB irradiation and oxidative stress and provide a new, more comprehensive methodology, taking into account both the influence of UVB irradiation and oxidative stress, for in vitro determination of SPF in cosmetic formulations.

## 1. Introduction

The skin is the largest organ in the body and performs many important functions, such as being a transport barrier against water loss and the entrance of toxic xenobiotics, defending against microbial pathogens, and providing a general protection against injuries [1,2]. Considering the complexity of the skin organ, in combination with presence of several external parameters that may compromise the skin integrity, such as oxidative stress [3] and exposure to ultraviolet radiation (UVR) [4], it is inherently challenging to assign a precise mechanism why a particular defective skin condition develops. Progress is being made on how to use topical or clinical therapies to reverse or alleviate the symptoms of defective or diseased skin; still, establishing the evidence of beneficial effects from various therapies in human populations remains elusive [2,3]. To approach this challenging topic and advance our general knowledge of how to maintain healthy skin, it is important to have access to reliable in vitro methods that allow for simple, fast, and inexpensive evaluation of relevant mechanisms responsible for defective skin and how topical therapies can be beneficially implemented. The aim of this work is to investigate the combined effect of UVR and oxidative stress on the skin barrier integrity of excised pig skin membranes in vitro by electrical impedance spectroscopy (EIS) measurements. Furthermore, the protective capacity of cosmetic sunscreen formulations against the combined assault from UVR and oxidative stress is examined with the aim to illustrate that the proposed in vitro methodology can be used to evaluate the sun protection factor (SPF) of cosmetic sunscreens.

In general, photons reaching the earth consist of 56% of infrared light (wavelengths 780–5000 nm), 39% of visible light (400–780 nm), and 5% of UVR (100–400 nm) [5]. Of the UVR reaching the earth’s surface, 95% is UVA (320–400 nm) and 5% is UVB (290–320 nm), while 0% of UVC (100–280 nm) is transmitted due to absorption from atmospheric ozone [5]. Solar irradiation is the main source of UVR, but in recent decades artificial sources have been developed. One reason for this is that artificial UV light can be taken advantage of for inducing beneficial effects of UVR, such as production of the vitamin D_3_ precursor in the epidermis and dermis, which occurs via photochemical action of UVB [5]. UVR is also used for treatment of skin conditions such as psoriasis, atopic dermatitis, vitiligo, and eczema [6,7]. Nonetheless, uncontrolled exposure of skin to UVR is a frequent health problem and it is well known that UVR cause damage to skin molecules, including DNA [8], and alter the mechanical integrity of the skin barrier [9], as well inducing indirect genotoxic effects mediated by reactive oxygen species (ROS) [3,10]. In particular, UVB irradiation can photochemically produce ROS radicals, such as the superoxide anion radical ($O_{2}{}^{•-}$) and hydroxyl radical (${}^{•}OH$) [4], which can cause significant oxidative damage of proteins and lipids of the skin barrier [8]. Taken together, when considering defective skin in general, it is difficult to disregard the fact that skin is a major target of UVR and oxidative stress from ROS.

The high exposure of skin towards oxidative stress is normally not a problem since the skin has a robust antioxidative system consisting of low molecular weight antioxidants [8,11] and antioxidative enzymes such as catalase, superoxide dismutase, glutathione peroxidase, peroxiredoxin, and heme oxygenase [3,12,13,14]. In particular, catalase is a principal enzyme of the antioxidative system of skin where it acts to detoxify hydrogen peroxide (H_2_O_2_) according to $2H_{2}O_{2}\;\overset{Catalase}{\to }2H_{2}O+O_{2}$ [12]. The importance of catalase in the skin organ is emphasized by its high expression in this tissue [10]; in particular, the expression of catalase is increasing in the skin towards the oxygen-rich atmosphere [13,14]. Further, the topical application of catalase of has been proposed to treat the inflammatory disease vitiligo, which is associated with reduced levels of catalase and increased concentrations of H_2_O_2_ in the epidermis of the depigmented skin site [15].

It is clear that acute, or chronic, exposure to combined assault from UVR and ROS can overwhelm the antioxidant defense mechanisms of the skin and contribute to the development of skin disorders, including skin cancer, skin aging, and dermatitis [3,4,8,10]. This issue is of particular relevance for the skin cosmetic field, where it is important to have simple and suitable methods for evaluating the performance of sunscreen formulations. At present, the only validated procedure for SPF determination involves in vivo measurements on human volunteers based on the generation of erythema from UVR, which is a biological end point mainly attributed to UVB irradiation [16]. More specifically, the in vivo method is based on the minimal erythema dose (MED), which is defined as the lowest dose of UVB irradiation that causes reddening and inflammation of the skin 24 to 48 h after exposure (i.e., the lowest UV dose that causes sunburn). The more sensitive an individual is to UVB exposure, the lower the MED of his/her skin and typical values are between approximately 15–150 mJ/cm^2^ [4]. From these measurements, the SPF value for a product is defined as the ratio of the MED measured with 2 mg/cm^2^ of applied sunscreen formulation to the MED corresponding to unprotected skin of the same subject [5]. In general, this in vivo method has the drawbacks of being expensive, time-consuming, and ethically questionable, besides being based on a subjective visual evaluation of skin redness. Therefore, there is considerable interest from the industry to develop new in vitro methods for SPF testing. Several in vitro techniques and protocols have been developed [17], but at present there is no broadly accepted method that can replace the in vivo method for SPF determination for labeling by authorities. Considering the strong connection between UVB irradiation, oxidative stress from ROS, and antioxidative enzyme function, as outlined above, it is clear that a more comprehensive methodology, taking into account these parameters, is highly relevant to develop. To approach this challenge, we have investigated the effect of UVB irradiation and oxidative stress on the electrical properties of excised pig skin membranes. In order to generate oxidative stress, the skin membrane was exposed to the ROS agent H_2_O_2_, which normally is detoxified by epidermal catalase (see above). Therefore, to simulate additional oxidative stress, the enzyme inhibitor NaN_3_ was employed to inhibit this detoxification process. This protocol is of particular biological relevance for the skin disorder vitiligo, which is associated with low levels of catalase and accumulation of H_2_O_2_ in the epidermis [15]. All experimental conditions were investigated with and without UVB exposure to secure proper reference values. Based on the results, a new simple in vitro methodology was developed, which was successfully verified by evaluating the protecting capacity of commercially available cosmetic sunscreen formulations with SPF values ranging from 10 to 70.

## 2. Materials and Methods

### 2.1. Materials

Hydrogen peroxide (H_2_O_2_, 30%, 9.8M), tablets for phosphate buffer saline (PBS, pH 7.4), sodium azide (NaN_3_), cetyl alcohol, mineral oil, and sodium dodecyl sulfate were purchased from Sigma Aldrich. All solutions were prepared from deionized water with resistivity of 18.2 Ωcm. Commercial sunscreens currently available on the market were selected on the basis of their SPF value (SPF 10, 20, 30, 50, and 70). The sunscreens contained different compositions of the same ingredients, without any antioxidants, which allows for a consistent comparison of the protecting capacity from each formulation under the present experimental conditions. The ingredients were: methylene-bis-benzotriazolyl tetramethylbutylphenol, ethylhexyl methoxycinnamate, diethylamino hydroxybenzoyl hexyl benzoate, ethylhexyl triazone, bisethylhexyloxyfenol methoxyphenyltriazine. A reference formulation with 0 SPF was prepared by mixing cetyl alcohol (8 wt.%), mineral oil (6 wt.%), sodium dodecyl sulfate (2 wt. %), and water (84 wt.%). This oil-in-water emulsion is referred to as cream (or 0 SPF) below.

### 2.2. Preparation of Skin Membranes 

Fresh pig ears were obtained from a local abattoir and stored at −80 °C until use. To prepare skin membranes, the frozen ears were thawed and rinsed with cold water and cut into strips with a scalpel. Hair was removed by an electrical clipper. From the tissue strips, skin from the inside of the ear was sliced out with a dermatome (TCM 3000 BL, Nouvag AG, Goldach, Switzerland), giving approximately 0.5 mm thick skin pieces. From the skin pieces, circular membranes with a diameter of 16 mm were punched out to fit the Franz cell that was used for impedance measurements. Membranes, not immediately used, were kept at −20 °C on a filter paper soaked in PBS and used within two weeks.

### 2.3. Narrowband UVB Irradiation

The source of radiation was a narrowband UVB bulb (Philips model PL-9 9W/01/2P) emitting photons with wavelengths between 306 and 316 nm, with a peak at 312 nm (without any meaningful radiation at other wavelengths). The narrowband UVB bulb was operated by a handheld phototherapy device from Philips connected to a Kernel system (model KN-4003BL, Kernel Medical Equipment Company, Xuzhou, China). The system was turned on at least 10 min prior to the experiment to ensure a stable radiation flux. Control measurements were performed to confirm that the irradiation output from this particular setup was in line with the specifications by employing a UV meter (UV-340A, Lutron Electronic Company, Taipei, Taiwan). The radiance was determined to 0.01 W/cm^2^ at a distance of 2 cm, which corresponds to the distance consistently used between the skin membrane and the light source. The narrowband UVB irradiation from this setup after 4 h to 6 h (as used herein) correspond to dosages between 144 and 216 J/cm^2^. It should be pointed out that these dosages are extremely high; considerably higher as compared to the naturally occurring solar UVB irradiation of any biological skin organ. For example, the annual UVB irradiation dosage ranges between roughly 30–130 J/cm^2^ depending on latitude [5]. However, UVB irradiation dosages above those that are physiologically normal was selected to amplify the effects on the skin barrier impedance properties. Further, it can be noted that the SC cohesion and mechanical integrity has been investigated after UVB dosages up to 800 J/cm^2^ [9].

### 2.4. Electrical Impedance Spectroscopy (EIS) Measurements of Skin Membranes

**Figure 1:**
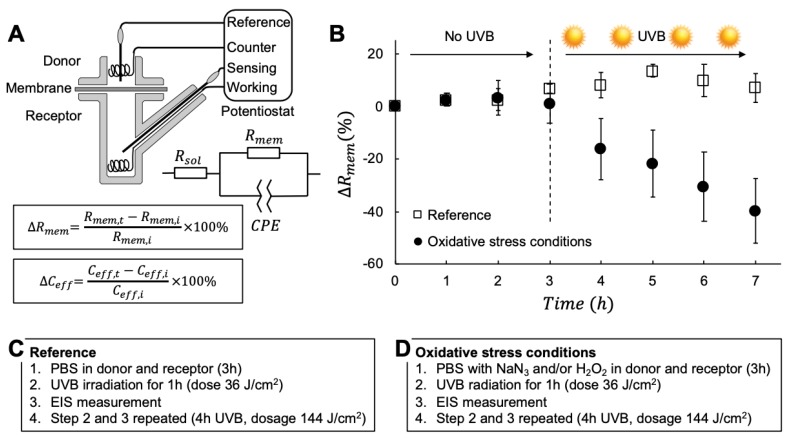
(**A**) Schematic representation of the 4-electrode EIS setup, equivalent circuit, and definitions of $\Delta R_{mem}$ and $\Delta C_{eff}$. Two platinum wires served as working and counter electrodes and two Ag/AgCl/3M KCl electrodes were used as sensing and reference electrodes. $R_{sol}$ is the resistance of the donor and receptor solution, $R_{mem}$ is the membrane resistance, and CPE is a constant-phase element used to derive the effective capacitance of the membrane, $C_{eff}$. (**B**) Representative data (average values ± SD, n = 3) from reference experiments with no oxidative stress parameters (i.e., neat PBS) and with oxidative stress conditions (in this case PBS containing 10 mM NaN_3_ and 1 mM H_2_O_2_). The impedance properties of the membranes were examined for 3h without UVB irradiation, followed by 4 h of UVB irradiation. The experimental procedures used to generate the data in (**B**) are specified in (**C**) and (**D**).

EIS measurements were performed with a four-electrode setup mounted in a Franz cell (Ø = 0.90 cm, V = 6 mL, PermeGear Inc., see Figure 1A). The electrodes were connected to a potentiostat from Ivium Technologies. Platinum wires were employed as working and counter electrodes, while Ag/AgCl/3M KCl electrodes (World Precision Instruments) were used as sensing and reference electrodes. All measurements were conducted under temperature control at 20 °C. The frequency range was from 0.1 Hz to 1 MHz with six frequencies per decade. The amplitude of the applied voltage was 100 mV. 

EIS is an established technique in electrochemistry that has gained attention as a tool for investigating the integrity and biophysical properties of biological tissues, such as the oral epithelium [18] or the skin organ [19]. In particular, EIS has been demonstrated to be a robust, simple and accurate method for characterization of skin cancer in human patients [19]. In addition, our previous work has also shown that EIS is very sensitive for detecting changes of the stratum corneum (SC) barrier properties of excised skin membranes in vitro, such as hydration-induced changes [20], which lead to significant changes of the resistive and capacitive currents [21]. Impedance in its simplest form describes the relation between voltage and current over a range of frequencies. Referring to Figure 1A, a measurement is performed by applying an alternating sinusoidal potential (voltage) between the working and counter electrodes so that the potential difference between the working and reference electrodes is equal to the set value of the potentiostat. The applied potential difference generates a response current between the counter and working electrodes, which is measured by the potentiostat. The impedance properties of the skin membrane contain both resistive and capacitive elements, which can be modeled with equivalent circuits of varying complexity. In this work, the EIS data were analyzed in accordance with an equivalent circuit consisting of a resistor (for solution resistance, $R_{sol}$), in series with a parallel combination of a resistor (for skin membrane resistor, $R_{mem}$) and a constant phase element (CPE) as shown in Figure 1A. This circuit is frequently used for analyzing skin impedance data [21,22,23]. The resistance values were obtained from the real part of the impedance in the frequency regions where the imaginary part gives minimum contribution to the total impedance. For $R_{sol}$, this region corresponds to high frequencies in the range of approximately 0.2–0.1 MHz. The corresponding frequency region for $R_{mem}$ occurs at low frequencies close to direct current (DC) where $R_{mem}=Z_{RE}-R_{sol}$. In this analysis, all data were normalized with the skin membrane area (0.64 cm^2^) to get units in Ohm cm^2^. The complex nature of skin membranes results in deviations from ideal properties, which has been recognized in several EIS studies on excised skin [21,22]. To account for this deviation it is common to include the empirical CPE element, which can be used to derive $C_{eff}$ [24]. For this, we followed a procedure in which the layers of epidermis are considered to have a distribution of time-constants [24]. The effective capacitance $C_{eff}$ was derived from the high frequency region from the imaginary impedance data by a procedure described in detail in previous studies [21,23]. The EIS experiments were designed to avoid the natural variability of individual skin membrane previously reported [21,23]. This was achieved by analyzing impedance data from individual membranes in terms of the change over time (*t*) of $R_{mem}$ and $C_{eff}$ from their initial (*i*) values according to:

(1) $$\Delta R_{mem}=\frac{R_{mem,t}-R_{mem,i}}{R_{mem,i}}\times 100\%$$

(2) $$\Delta C_{eff}=\frac{C_{eff,t}-C_{eff,i}}{C_{eff,i}}\times 100\%$$

### 2.5. Experimental Design

**Figure 2:**
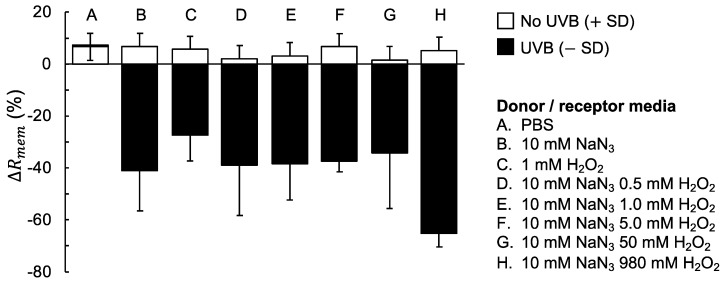
Summary of $\Delta R_{mem}$ (%) after 3 h without UVB irradiation and 4 h of total UVB irradiation (corresponding to 144 J/cm^2^) in combination with different stress parameters present in both the donor and receptor media. Data show average values (n = 3) with error bars showing either +SD (without UVB) or −SD (with UVB); n = 6 for A and n = 2 for F and G.

**Figure 3:**
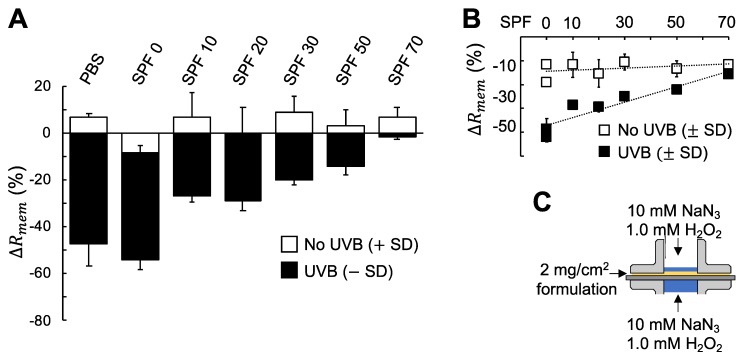
(**A**) Summary of $\Delta R_{mem}$ (%) after 6 h UVB irradiation (216 J/cm^2^) and protection from topically applied sunscreen formulations. PBS with 10 mM NaN_3_ and 1 mM H_2_O_2_ was included as control, without and with UVB irradiation. Data show average values (n = 3) with error bars showing either +SD (without UVB) or −SD (with UVB). (**B**) $\Delta R_{mem}$ as a function of SPF value without and with UVB irradiation. The coefficient of determination for the regression line corresponding to the $\Delta R_{mem}$ after UVB irradiation was r^2^ = 0.87. (**C**) Schematic illustration of the experimental setup with presence of 10 mM NaN_3_ and 1 mM H_2_O_2_ in the donor and receptor media.

**Figure 4:**
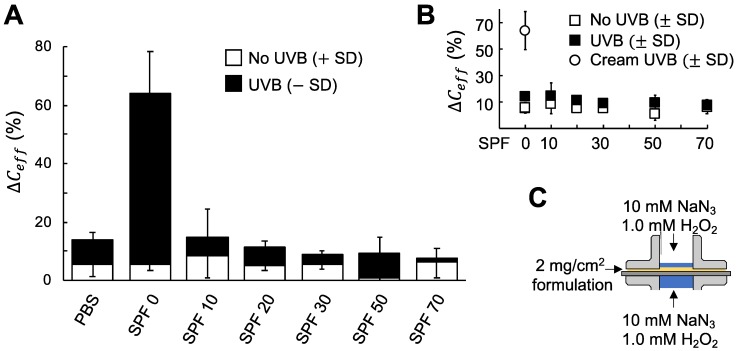
(**A**) Summary of $\Delta C_{eff}$ (%) after 6 h UVB irradiation (216 J/cm^2^) and protection from topically applied sunscreen formulations. PBS with 10 mM NaN_3_ and 1 mM H_2_O_2_ was included as control, without and with UVB irradiation. Data show average values (n = 3) with error bars showing either +SD (without UVB) or −SD (with UVB). (**B**) $\Delta C_{eff}$ as a function of SPF value without and with UVB irradiation. (**C**) Schematic illustration of the experimental setup with presence of 10 mM NaN_3_ and 1 mM H_2_O_2_ in the donor and receptor media.

To investigate the combined effect of UVB irradiation and oxidative stress from H_2_O_2_ and NaN_3_ on the electrical properties of skin, the following experimental design was used (see Figure 1). The receptor chamber of the Franz cell was filled with degassed PBS solution, after which the skin membrane was mounted and kept without donor solution and for 1h to reach an initially stable state in terms of temperature and hydration. Next, the different stress agents (i.e., NaN_3_ and/or H_2_O_2_) were added to both the donor and receptor solution (control experiments were performed without NaN_3_ and H_2_O_2_). Then, EIS measurements were performed every hour for 3 h without UVB irradiation in order to establish reference values of the effect of the oxidative stress agents by themselves (without UVB). Subsequently, the membrane was irradiated with UVB for 1h, corresponding to a dose 36 J/cm^2^, in the presence of oxidative stress conditions (control experiments were performed with NaN_3_ and H_2_O_2_ and without UVB irradiation). The donor and receptor media were always present during irradiation of UVB to assure full action of the oxidative stress agents and to avoid drying of the skin membrane. It should be pointed out that the heat generated by the UVB lamp was counteracted by cooling the system to assure a constant temperature of 20 °C. After UVB exposure, the EIS measurements were conducted again. This cycle was repeated to achieve 4 h in total of UVB exposure time (see Figure 2), which corresponds to a dosage of 144 J/cm^2^. In the evaluation of the sunscreen formulations it was decided to prolong the UVB exposure time to 6 h in total (see Figure 3 and Figure 4), which corresponds to a dosage of 216 J/cm^2^. All measurements were performed in triplicates (n = 3) under the following experimental conditions:

- Exposure to UVB irradiation (without additional oxidative stress from H_2_O_2_ and NaN_3_)
- Exposure to UVB irradiation with presence of 10 mM NaN_3_ in the donor and receptor solution
- Exposure to UVB irradiation with presence of 1 mM H_2_O_2_ in the donor and receptor solution
- Exposure to UVB irradiation with presence of 10 mM NaN_3_ and varying concentrations of H_2_O_2_ (i.e., 0.5, 1.0, 5.0, 50, 980 mM H_2_O_2_) in the donor and receptor solution
- Exposure to UVB irradiation with presence of topically applied sunscreen formulation with SPF varying between 0, 10, 20 30, 50, 70.

For case E, a dose of 2 mg/cm^2^ of sunscreen formulation was applied topically (i.e., standard dose). Next, approximately 50 µl of PBS containing 10 mM NaN_3_ and 1 mM H_2_O_2_ was added on top of the formulation in the donor chamber as a source of oxidative stress. In addition, in this manner the possibility of drying of the membrane was avoided, which otherwise may occur in the case of surface regions with low or inadequate formulation coverage. Similarly, a receptor solution containing 10 mM NaN_3_ and 1 mM H_2_O_2_ in PBS was used in these experiments (i.e., case E).

In general, it should be pointed out that this study design includes control experiments, where the effect of the stress agents by themselves on each individual skin membrane is investigated for 3h without exposure to UVB, followed by 4 h or 6 h with exposure to UVB irradiation. In other words, this design enables us to distinguish between the effect of NaN_3_ and/or H_2_O_2_ per se and the combined effect of these oxidative stress agents and UVB exposure. In addition, control experiments without exposure to neither UVB nor oxidative stress were performed and included as reference.

### 2.6. Histology and Microscopy 

Light microscopy was employed to investigate the combined effect of UVB irradiation and oxidative stress conditions on the macroscopic features of the skin membrane integrity. For these experiments, skin membranes were immersed in PBS solution containing 10 mM NaN_3_ and 1 mM H_2_O_2_ and exposed to UVB irradiation for 5h (corresponding to a dose of 180 mJ/cm^2^). As a reference, the skin membranes were immersed in PBS solution containing 10 mM NaN_3_ and 1 mM H_2_O_2_ for 5 h without UVB treatment. Next, the skin membranes were prepared by a standard staining procedure with hematoxylin and eosin. After the staining procedure, histological sections of 5 µm thickness were sliced from paraffin embedded samples and fixed with 10% formaldehyde, before light microscopy imaging (Leica DM500 light microscopy CH-9435). 

### 2.7. Statistical Analysis

The differences in the mean values of $\Delta R_{mem}$ or $\Delta C_{eff}$ between groups were analyzed with 2-tailed two-sample *t*-tests, assuming equal variance, and *p*-values lower than 0.05 was considered as statistically significant.

## 3. Results

### 3.1. A New Protocol for Investigating UVB and Oxidative Stress with Electrical Impedance Spectroscopy

The aim of this work was to investigate the combined effect of UVB irradiation and oxidative stress on the electrical properties of the skin barrier and to develop a new methodology for evaluating the SPF of cosmetic sunscreen formulations. To achieve this, we developed a new simple in vitro method based on EIS measurements of excised pig skin membranes. The setup is presented in Figure 1A, together with the model circuit used to analyze the data (a detailed description of the analytical procedure is given elsewhere [21,23]). To illustrate the general experimental procedure, we present representative data in Figure 1B from reference experiments (Figure 1C) and experiments with oxidative stress conditions (Figure 1D).

A first conclusion from the results in Figure 1B is that $\Delta R_{mem}$ remains more or less unaffected for the first 3h when no UVB irradiation occurs. Notably, this conclusion is also true for $\Delta R_{mem}$ corresponding to the membranes exposed to oxidative stress conditions for the first 3h in Figure 1B (without UVB exposure). In fact, this observation is valid for all experimental conditions studied herein, irrespective of presence of the catalase inhibitor NaN_3_ and/or the ROS agent H_2_O_2_ (see below). The second conclusion from Figure 1B is that exposure to 4 h of UVB irradiation does not influence $R_{mem}$ when no additional oxidative stress parameters are present (i.e., reference data in Figure 1B). This is a striking finding considering that the UVB dosage is extremely high (i.e., 144 J/cm^2^) and shows that $\Delta R_{mem}$ is virtually unaffected by UVB irradiation alone. This is in contrast to the combined exposure of UVB and oxidative stress from NaN_3_ and/or H_2_O_2_, which results in a clear decrement of $\Delta R_{mem}$ (e.g., oxidative stress data in Figure 1B). 

### 3.2. The Combined Effect of UVB Irradiation And Oxidative Stress on the Skin Barrier Electrical Properties

To evaluate the combined effect of UVB irradiation and oxidative stress in more detail we performed additional experiments, in accordance to the general procedure illustrated in Figure 1. The results from these experiments are presented in Figure 2 where $\Delta R_{mem}$ after 3 h without UVB exposure are compared with $\Delta R_{mem}$ after 4 h of UVB irradiation. 

The results from the experiments without UVB irradiation presented in Figure 2, (i.e., No UVB from all treatments) clearly show that exposure to 10 mM NaN_3_ and/or H_2_O_2_ at concentrations of 0.5, 1.0, 5.0, 50 and 980 mM does not markedly influence $\Delta R_{mem}$. In fact, the results corresponding to these treatments, without UVB irradiation, are similar as compared to neat PBS solution (*p*-values > 0.05 between groups in all cases). This important observation proves that NaN_3_ and H_2_O_2_ do not influence $\Delta R_{mem}$ by themselves under the present experimental conditions. The next clear observation in Figure 2 is that $\Delta R_{mem}$ is drastically decreased after exposure to a combination of UVB irradiation and either NaN_3_ or H_2_O_2_, which is in contrast to the case of UVB irradiation with neat PBS. Further, the most drastic decrease of $\Delta R_{mem}$ was observed for the highest concentration of H_2_O_2_, which is perhaps not surprising considering that 980 mM H_2_O_2_ is a very high concentration. In summary, it is clear that $\Delta R_{mem}$ corresponding to case A (i.e., neat PBS) after UVB irradiation is significantly less affected as compared to $\Delta R_{mem}$ corresponding to all other treatments (i.e., cases B, C, D, E, F, G, and H) with *p*-values ranging between 0.000 and 0.008 based on two-sample *t*-tests between groups. In addition, $\Delta R_{mem}$ corresponding to treatment in 980 mM H_2_O_2_ (i.e., case H) is significantly more reduced as compared to $\Delta R_{mem}$ corresponding to cases C, E, and F (p-values between 0.006 and 0.035), while $\Delta R_{mem}$ from cases B, D, and G can be considered to be similar to case H (i.e., *p*-values above 0.05). 

Taken together, the main result from Figure 2 is that the presence of NaN_3_ and/or H_2_O_2_, together with UVB irradiation, induces a significant decrease of $\Delta R_{mem}$. On the other hand, there is no clear dose response with respect to increasing the concentration of H_2_O_2_ in the range between 0.5 and 50 mM (in the presence of 10 mM NaN_3_). Based on these results, it is clear that any of the oxidative stress conditions can be used, together with an acute dose of UVB irradiation, in order to induce a significant decrease of $\Delta R_{mem}$. However, we decided to include both NaN_3_ (10 mM) and H_2_O_2_ (1 mM) in the treatment protocol for further experiments. The reason for this was to simulate oxidative stress by simultaneous inhibition of catalase (i.e., by NaN_3_) and to assure that the treatment included a known source of ROS (i.e., from H_2_O_2_). Also, H_2_O_2_ at high concentrations can be practically challenging due to formation of gas bubbles, which potentially may influence the measurement (for example if gas is trapped below the membrane). Thus, 1 mM H_2_O_2_ is a reasonable concentration in this regard and together with 10 mM NaN_3_ a significant decrease of $\Delta R_{mem}$ is ensured after and UVB irradiation (Figure 2E). 

### 3.3. A New Method to Evaluate Sun Protection Factor (SPF) based on Electrical Impedance Spectroscopy (EIS)

Next, we investigated the possibility to employ EIS on excised skin membranes in vitro to evaluate the protecting capacity of cosmetic sunscreen formulations with varying degrees of SPF. For this, it was decided to employ experimental conditions that lead to a clear and drastic reduction of $\Delta R_{mem}$, which is fulfilled by simulating oxidative stress with 1 mM H_2_O_2_ and 10 mM NaN_3_, together with UVB irradiation (see Figure 1B and Figure 2E). To evaluate the protection from this harsh experimental condition, a standard dose of sunscreen formulation (2 mg/cm^2^) was topically applied on the skin membrane. To establish reference values of $\Delta R_{mem}$, the membranes were initially examined with EIS for 3 h without UVB irradiation in PBS containing 1 mM H_2_O_2_ and 10 mM NaN_3_. Thereafter, sunscreen protected membranes were exposed to UVB irradiation for 6 h in total (corresponding to a dosage of 216 J/cm^2^). As controls, both neat PBS (without any topical formulation) and a cream with 0 SPF were included in these experiments (with presence of 1 mM H_2_O_2_ and 10 mM NaN_3_ and UVB irradiation). Further, it can be noted that the exposure time for the UVB irradiation was extended with 2 h (from 4 h to 6 h) to obtain more challenging conditions. Other than these modifications, the experimental protocol was kept the same as for previous measurements in accordance with the procedure described in Figure 1. The results from these experiments are presented in Figure 3. 

The results in Figure 3 illustrate, once again, that the combination of UVB irradiation and oxidative stress from NaN_3_ and H_2_O_2_ results in a significant decrease of $\Delta R_{mem}$, which is not observed in the case of only exposure to NaN_3_ and H_2_O_2_ (i.e., the data corresponding to No UVB in Figure 3). Further, by comparing the results in Figure 2 and Figure 3 it is possible to conclude that the treatment of the skin membrane with 6 h of UVB irradiation, under immersion in PBS containing 10 mM NaN_3_ and 1.0 mM H_2_O_2_, results in $\Delta R_{mem}\;$ = −48 ± 9% (Figure 3), which is in line with the results in Figure 2A from similar treatment, but after a shorter exposure time of 4 h UVB, where $\Delta R_{mem}\;$ = −38 ± 14. Moreover, the results in Figure 3 show that increased SPF value results in a sequentially increased capacity to retain the integrity of the membrane, as judged by the fact that $\Delta R_{mem}$ is less affected for higher SPF values. This conclusion is clearly supported by the regression analysis presented in Figure 3B for the UVB treated samples (r^2^ = 0.87). It should be noted that the stress conditions were identical for all these experiments and that the only parameter that was varied was the SPF value of the sunscreen. In other words, the results in Figure 3 clearly illustrate that the proposed methodology successfully allows for evaluation of sunscreen formulations with different SPF values. 

The EIS data corresponding to the experiments presented in Figure 3 were analyzed in terms of the effective capacitance of the skin membranes ($\Delta C_{eff}$) to obtain a more complete picture of the protecting capacity of the sunscreen formulations. The results from this analysis are summarized in Figure 4.

Interestingly, Figure 4A shows that $\Delta C_{eff}$ remained less affected by exposure to the combination of UVB irradiation and oxidative stress from NaN_3_ and H_2_O_2_, as compared to $\Delta R_{mem}$ presented in Figure 3 from the corresponding skin membranes. In general, $\Delta C_{eff}$ increased about 10% after the UVB irradiation treatment (Figure 4A,B). However, this increase cannot be distinguished from the initial increase of $\Delta C_{eff}$, which is likely due to skin hydration leading to increased skin membrane capacitance [21]. Therefore, the data of $\Delta C_{eff}$ in Figure 4 can be regarded as more or less constant, irrespective of SPF value (i.e., no correlation between these parameters as shown in Figure 4B). The only treatment that resulted in a statistically significant change of $\Delta C_{eff}$ was UVB irradiation with topical cream with 0 SPF (*p*-values between 0.002–0.008 when comparing $\Delta C_{eff}$ corresponding to this case with all other treatments, see Figure 4A,B). Considering that the composition of the cream with 0 SPF was different as compared to the commercially sunscreen products (see above), it is possible that some specific ingredient of this cream induces the observed change of $\Delta C_{eff}$ after UVB irradiation. However, it should be pointed out that the ingredients of this cream are commonly used in commercial skin care products (without sunscreen protection), which implies that this observation is of general relevance. Except for this significant result, no differences in $\Delta C_{eff}$ corresponding to different treatments were observed (i.e., *p*-values above 0.05). 

### 3.4. UVB Irradiation in the Presence of Oxidative Stress Conditions leads to Substantial Damage of the Skin Membrane 

**Figure 5:**
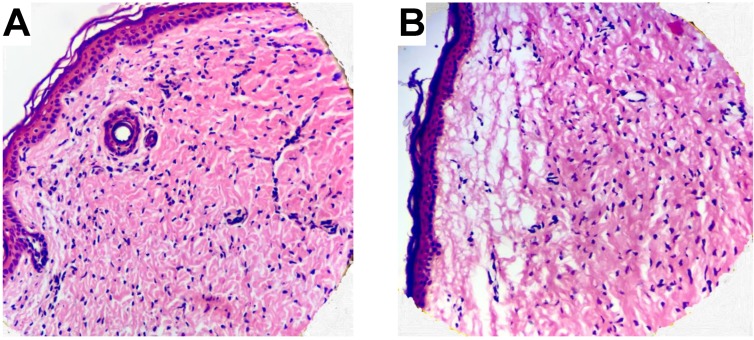
Excised pig skin membrane soaked in 10 mM NaN_3_ and 1 mM H_2_O_2_ for 5 h without (**A**) and with (**B**) exposure to UVB irradiation (dosage corresponding to 180 J/cm^2^).

Finally, light microscopy imaging was performed to investigate the combined effect of UVB and oxidative stress from NaN_3_ and H_2_O_2_ on the macroscopic integrity of the skin membrane. For this, the skin membrane was treated by the identical procedure as during the EIS measurements by exposing the membrane to UVB irradiation for 5 h (corresponding to a dosage of 180 J/cm^2^), while being immersed in PBS solution containing 10 mM NaN_3_ and 1 mM H_2_O_2_. In addition, another membrane was immersed in PBS solution containing 10 mM NaN_3_ and 1 mM H_2_O_2_ without UVB irradiation as reference. In other words, these experimental conditions correspond to the data presented in Figure 1B (with the exception of 5 h exposure time) under oxidative stress conditions without UVB (0–3 h) and with UVB irradiation (3–7 h). The results from these experiments are presented in Figure 5. 

By comparing the histological images in Figure 5, it is clear that the combination of UVB irradiation and oxidative stress from NaN_3_ and H_2_O_2_ results in significant tissue damage and breakdown of the skin membrane integrity (Figure 5B). In particular, the epidermal layers beneath the SC are significantly damaged, while the staining of the SC barrier is clearly altered (Figure 5B). Taken together, it is likely that the status of the tissue sample presented in Figure 5B corresponds to an impaired skin barrier towards molecular transport. This conclusion is in line with the impedance results presented in Figure 1B showing a drastic decrease of $\Delta R_{mem}$, which is initiated by UVB irradiation of the skin membrane in the presence of NaN_3_ and H_2_O_2_. On the other hand, $\Delta R_{mem}$ does not change during the first hours (Figure 1B, no UVB irradiation). This is supported by the image in Figure 5A showing that the membrane remains relatively intact with proper skin barrier towards molecular transport after treatment with NaN_3_ and H_2_O_2_ (without UVB irradiation).

## 4. Discussion

The skin barrier is directly exposed to UVR from sunlight and the oxygen-rich external environment; it is therefore a major target of photochemically damaging processes and oxidative stress from ROS. The epidermal antioxidant defense mechanisms can be depleted by acute or chronic UVR exposure and, together with oxidative stress, make the skin susceptible to various skin disorders [3,4,8,10]. To advance the understanding of this complex topic, it is important to have access to simple, fast, and inexpensive methods that allow for reliable evaluation of these stress parameters on the skin structure and function. However, at present there is a lack of in vitro methods that take into account the combined assault from UVR and ROS on the skin barrier integrity. Here, we introduce a new methodology to investigate the collective effect of these parameters by EIS measurements on excised pig skin in vitro (see Figure 1). To generate oxidative stress, the skin is exposed to the ROS H_2_O_2_, while the enzyme inhibitor NaN_3_ is used to inactivate the antioxidative enzyme catalase. The combined exposure of UVB, H_2_O_2_, and NaN_3_ is of particular biological relevance for the skin depigmentation disorder vitiligo, which is associated with low levels of catalase and accumulation of H_2_O_2_ in the epidermis [15].

### 4.1. The Skin Membrane Electrical Resistance Is not Influenced by UVB Irradiation 

In general, the observed effects of the skin membrane electrical resistance ($R_{mem}$) are clear and can be rationalized in terms of the skin barrier towards electrical current. A relevant starting point for discussion of the present results is to consider the origin of the electrical resistive properties of the skin membrane. Several studies have proposed that ions, which represent the charge carriers of an electrical current, are primarily transported, and hence distributed, in the extracellular domains of the SC barrier [25,26]. The extracellular matrix consists primarily of stacked lipid lamellar structures and represents the only continuous element across the skin barrier, which therefore has to be permeated by ions to allow for electric currents [2]. In addition, there is strong evidence that tight junctions (TJs) represent a significant barrier towards diffusion of ions and low molecular weight molecules [27]. TJs are multiprotein structures that seal the intersections of adjacent keratinocytes in the stratum granulosum (SG), which is found below the SC [27]. Even though the assembly of these structures represent a robust barrier, it is a striking observation that $\Delta R_{mem}$ remains virtually unaffected after exposure to an extreme dosage of UVB irradiation of 144 J/cm^2^ (see Figure 2A). In fact, this dosage is about 100–1000 times higher than typical values of MED (minimal erythema dose) for human patients (e.g., 0.4–1.2 J/cm^2^ [28] or 0.1–0.8 J/cm^2^ [29]). This shows that the electrical resistance of the skin barrier is largely insensitive to UVB exposure per se under the conditions investigated herein (Figure 2A), implying that the macroscopic skin barrier remains intact. Similarly, a previous study showed that the stiffness of SC, which is mainly controlled by the keratin filaments of the corneocytes, remained virtually constant after exposure to an extreme UVB dosage of 800 J/cm^2^ [9]. It is important to note that there is a lag time, corresponding to days, between an acute UVB assault and the biological response that leads to inflammation and defective skin barrier integrity [28,29,30]. Therefore, there is no contradiction between the present results and previous reports showing that the SC barrier becomes reduced several days after an acute dose of UVB irradiation [30]. For example, based on measurements of the transepidermal water loss of hairless mice (TEWL) it has been shown that the SC barrier is significantly weakened three days after an acute UVB irradiation (0.15 J/cm^2^) [30]. However, it was also concluded that the TEWL values were not statistically different after one or two days following acute UVB treatment, as compared to the untreated control [30]. Taken together, it is reasonable to suggest that an acute and extreme dosage of UVB irradiation does not result in an immediate impairment of the skin barrier integrity, which explain why there is no observed significant reduction of $\Delta R_{mem}$ after UVB irradiation in the present work (Figure 2A). 

### 4.2. The Combined Effect of UVB Irradiation and Oxidative Stress Results in a Significant Decrease of the Skin Membrane Electrical Resistance

The second clear observation is that $\Delta R_{mem}$ is significantly reduced after exposure to a combination of UVB irradiation and H_2_O_2_ and/or NaN_3_ (Figure 2B–H), which implies that the integrity of the skin barrier is compromised. In particular, it is likely that ROS radicals, such as the superoxide anion radical ($O_{2}{}^{•-}$) and hydroxyl radical (${}^{•}OH$) [4], are generated by the these treatments (Figure 2B–H). It is known that these radicals cause oxidative damage of the proteins and lipids comprising the skin barrier [8]. In other words, it is probable that $\Delta R_{mem}$ is reduced due to alterations of the lipids of the lamellar matrix of the SC and the proteins of the TJs in SG, which effectively can introduce defective regions where ions can be transported with low resistance across the skin barrier. This is in line with the observed signs of macroscopic tissue damage of the epidermis after exposure to UVB radiation and oxidative stress (Figure 5B). Notably, there is no clear dose response with respect to an increasing concentration of H_2_O_2_ (Figure 2), implying that the induced conductive pathways across the skin barrier do not increase in size as a function of H_2_O_2_ concentration. Speculatively, this can, for example, be explained by breakdown of some structural element of the skin barrier, which is finite and therefore only leads to a finite decrease of $\Delta R_{mem}$, independent of the concentration of H_2_O_2_ (between 0.5 and 50 mM). 

It has been reported that a UVB irradiation dosage of 2.8 J/cm^2^ caused a significant decrease of the catalase activity in mice, as compared to the non-irradiated control [31]. Therefore, we hypothesized that UVB irradiation would lower the removal rate of H_2_O_2_ and lead to oxidative damage of the skin barrier from UVB irradiation alone, without any supplementary H_2_O_2_. However, the fact that $\Delta R_{mem}$ remains constant after UVB irradiation (see Figure 2A) implies that the concentration of naturally occurring H_2_O_2_ is too low to cause any detectable oxidative damage of the skin barrier from the present impedance measurements. Considering this, a question arises regarding the mechanism leading to the observed significant decrease of $\Delta R_{mem}$ after treatment with NaN_3_, without any additional H_2_O_2_ (see Figure 2B). If the protocol for UVB irradiation and NaN_3_ exposure used herein are equally efficient in terms of inhibiting catalase, these experiments are expected to generate similar values of $\Delta R_{mem}$, which they do not (Figure 2A,B). Speculatively, these findings may be due to the fact that UVB irradiation does not inhibit epidermal catalase as efficiently as NaN_3_, or that UVB irradiation induces some unknown photochemical damage of the skin barrier in the presence of NaN_3_.

### 4.3. Comprehensive Evaluation of the Protecting Capacity of Sunscreen Formulations against the Combined Assault Of UVB Irradiation And Oxidative Stress 

The third main finding of this work is that the significant decrease of $\Delta R_{mem}$ can be minimized by topical application of sunscreen formulation, which protects against the combined assault from UVB radiation and oxidative stress (Figure 3A). The results clearly demonstrate that the protecting effect of the applied sunscreen correlate well with the degree of SPF (Figure 3B). This new methodology is promising as a simple and relatively fast in vitro method for assessment of sunscreen cosmetic formulations. 

One benefit of analyzing the impedance data in terms of resistance and capacitance can be illustrated by comparing the results in Figure 3 ($\Delta R_{mem}$) and Figure 4 ($\Delta C_{eff}$). In particular, the change of $\Delta C_{eff}$ in Figure 4B, after UVB irradiation and exposure to oxidative stress, is relatively weak as compared to the corresponding change of $\Delta R_{mem}$ in Figure 3B. This is in contrast to the change of $\Delta C_{eff}$ in Figure 4C (i.e., treatment with cream with 0 SPF) and the corresponding value of $\Delta R_{mem}$ in Figure 3B, which both changes significantly. In other words, both treatments lead to drastic decreases of $\Delta R_{mem}$, but it is only the cream treatment that significantly alters $\Delta C_{eff}$. To explain this, it is relevant to understand the source of the capacitive currents of the skin membrane, which is usually attributed to the dielectric nature of lipid lamellar structures that can build up capacitive currents by blocking transport of ions [21,32]. Thus, if $\Delta C_{eff}$ reflects alterations of the lipid lamellar matrix of the SC barrier, then these domains are significantly affected by the cream treatment, after UVB irradiation (Figure 4C). However, application of the cream alone, without UVB irradiation, does not affect the SC lipids in the same manner, as judged from the nearly constant value of $\Delta C_{eff}$ observed in Figure 4A. This implies that the increase of $\Delta C_{eff}$ (Figure 4C) is most likely related to the combination of UVB irradiation and some component of the oil-in-water emulsion. Interestingly, pretreatment with mineral oil, before UVB therapy, has been shown to significantly increase the plaque clearance in psoriasis, especially in severe psoriasis, where the scaling and infiltration were significantly improved [33]. Thus, it is possible that the significant increase of $\Delta C_{eff}$ observed in Figure 4C is related to presence of mineral oil, in combination of UVB treatment. However, it is difficult to rule out that this increase effect equally well could be due to the presence of cetyl alcohol or sodium dodecyl sulfate and UVB radiation. Even though the combined analysis of $\Delta R_{mem}$ and $\Delta C_{eff}$ is not fully conclusive, this complementary examination definitely provides a more comprehensive picture of the effects of various treatments on the skin membrane electrical properties.

## 5. Conclusions

The aim of this work was to investigate the combined effect of UVB radiation and oxidative stress on the electrical properties of the skin barrier. For this, EIS was employed to characterize changes of the skin membrane resistance ($\Delta R_{mem}$) and effective capacitance ($\Delta C_{eff}$) of excised pig skin. In particular, changes of skin electrical impedance induced by exposure to UVB irradiation in the presence, or absence, of oxidative stress parameters were investigated (see Figure 1). The oxidative stress was induced by adding H_2_O_2_ as a source of ROS, while NaN_3_ was supplemented to inhibit the antioxidative enzyme catalase, which is naturally present in epidermis (see Figure 2). The main conclusions from this work can be summarized by following points:

- $\Delta R_{mem}$ and $\Delta C_{eff}$ remain largely unaffected by exposure to an extreme dosage of UVB irradiation (Figure 1B and Figure 2A and PBS control in Figure 3 and Figure 4).
- If no UVB irradiation is applied to the skin membrane, $\Delta R_{mem}$ and $\Delta C_{eff}$ are not significantly affected by exposure to oxidative stress from 10 mM NaN_3_ and H_2_O_2_ in concentrations ranging between 0.5 mM and 980 mM (data without UVB irradiation in Figure 1, Figure 2, Figure 3 and Figure 4). This conclusion is supported by the relatively intact skin integrity observed by microscopy imaging after exposure to oxidative stress conditions (Figure 5A).
- The combined assault from UVB irradiation and oxidative stress conditions results in a significant decrease of $\Delta R_{mem}$ (Figure 2). This conclusion is supported by the severe tissue damage observed by microscopy imaging after exposure to UVB irradiation in the presence of oxidative stress conditions (Figure 5B).
- A new methodology is presented, based on EIS measurements, which successfully allows for the evaluation of the protecting capacity from topical sunscreen formulations against the combined assault from UVB irradiation and oxidative stress conditions (Figure 3 and Figure 4).
- demonstration of the proposed methodology for in vitro testing of cosmetic sunscreen formulations with varying SPF values is presented, showing good correlation between $\Delta R_{mem}$ and SPF values (Figure 3B), while $\Delta C_{eff}$ is shown to be virtually constant irrespective of SPF value (Figure 4B).

Finally, it should be pointed out that there are many possibilities to adjust the protocol for optimization with respect to the research question that is addressed. For example, screening for beneficial and protecting effects from various relevant compounds, such as anti-inflammatory lipid species, vitamin C, vitamin E, ascorbate, tocopherol, and polyphenols [11,31,34], to mention a few, could be investigated with the proposed methodology. Further, the results from this study invite the development of novel skin sensors based on EIS for the detection of skin tissue damage due to exposure to UVB irradiation and oxidative stress.
